# Supplementary material for: Distinct Expression of Inflammatory Features in T Helper 17 Cells from Multiple Sclerosis Patients
Source: Cells. 2019 Jun 4;8(6):533. doi: 10.3390/cells8060533 (PMC6628300; doi:10.3390/cells8060533)

Supplementary figure S1. The activity of MS disease does not modulate IL-1R1 expression on differentiated Th17 cells

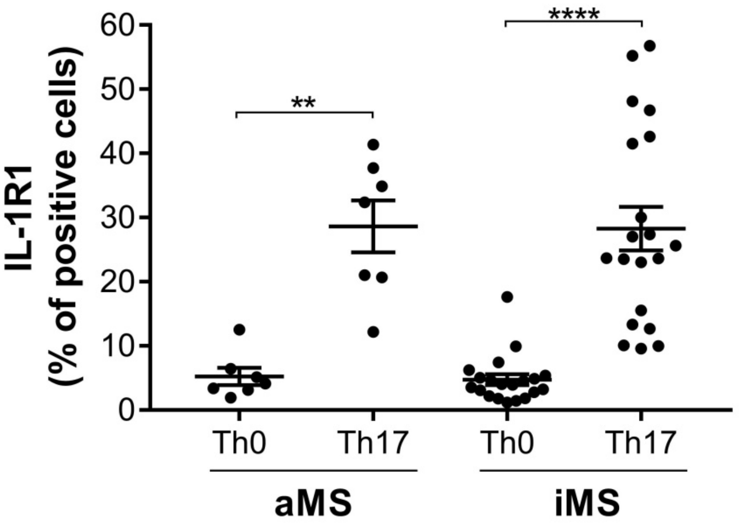

Supplementar figure S2. The sinergy between Th17 polarizing cytokines contributes to IL-1R1 expression on Th17 cells.

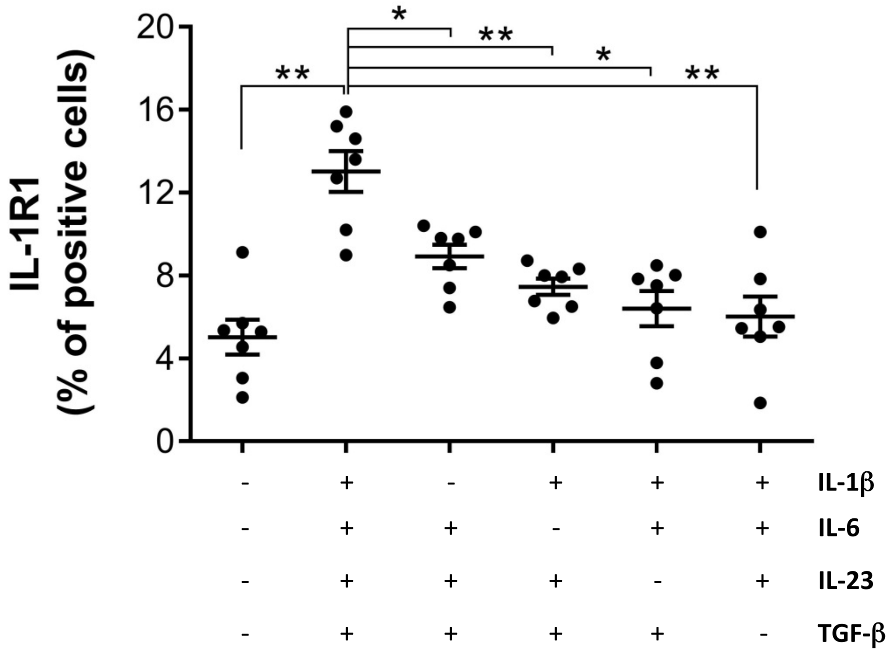

Supplement: Supplementary file 1 [file cells-08-00533-s001.pdf]
